# Health information provision, health knowledge and health behaviours: Evidence from breast cancer screening

**DOI:** 10.1016/j.socscimed.2020.113505

**Published:** 2020-11

**Authors:** Peter Eibich, Léontine Goldzahl

**Affiliations:** a Max Planck Institute for Demographic Research, Konrad-Zuse-Str. 1, 18057, Rostock, Germany; b Health Economics Research Centre, Nuffield Department of Population Health, University of Oxford, UK; c EDHEC Business School, France

**Keywords:** Information, Knowledge, Health behaviour, Mediation analysis, Mammography, Breast cancer, Eurobarometer

## Abstract

Many public health interventions aim to provide individuals with health information on the consequences of behaviours such as smoking, alcohol consumption or preventive care use, with the intention of changing health behaviour through better health knowledge. This paper examines whether the provision of health information in organised breast cancer screening programs affects mammography utilisation via changes in health knowledge. We use unique data on 10,610 European women from the Eurobarometer survey collected in 1997/1998, and we exploit variation in the availability and coverage of organised breast cancer screening programs for causal identification in a difference-in-differences design. We find that health information provision improves health knowledge. Yet, these changes in health knowledge had little to no effects on mammography utilisation in the overall population. Our findings imply that health information provision contributes little to health behaviour change. Although screening programs are effective at increasing preventive care use, their effect can be attributed almost entirely to factors other than health knowledge.

## Highlights

• We estimate a difference-in-differences model and conduct a mediation analysis.
• We exploit variation in screening program existence and eligibility ages in Europe.
• Screening program eligibility improves health knowledge about breast cancer.
• Changes in health knowledge contribute little to screening program uptake.
• Health information provision has a stronger impact on less educated women.

## 1 Introduction

Engagement in health preserving behaviours, such as exercising, smoking cessation or cancer screening, are considered major behavioural changes that can lower the burden of non-communicable diseases, such as cardiovascular diseases or cancer representing 71% of all deaths globally (WHO, 2019). The lack of engagement in such health behaviours is often explained by the imperfect knowledge on their benefits, as well as systematic underestimation of the marginal productivity of medical care (Arrow, 1963; Kenkel, 1990, 1991). Health authorities disseminate information through mass informational campaigns to promote health behaviour changes. The underlying assumption is that additional information would improve individuals' knowledge leading them to reconsider the costs and benefits of engaging in health preserving behaviour. Nevertheless, the impact of these information policies on health knowledge and subsequent behaviours are rarely evaluated.

In this paper, we examine whether providing information on breast cancer screening shapes recipients' knowledge and affects subsequent screening take-up. Our identification strategy of the effect of health information on health knowledge relies on the informational shock triggered by variation in eligibility for breast cancer screening programs in Europe. Eligible women receive an invitation letter containing information on breast cancer, screening procedures and the benefits of screening. We take advantage of variation in availability of breast cancer screening programs between European countries as well as variation in eligibility ages in a difference-in-differences (DID) design to measure the causal impact of information on women's knowledge of breast cancer screening and treatment using data from the Eurobarometer surveys from 1997 to 1998. A mediation analysis allows us to quantify how much of the effect of screening program eligibility on breast cancer screening operates through changes in knowledge.

Our paper contributes to a broader literature in psychology and economics on how health information influences health behaviours. Several models in psychology illustrate how health information provision leads to behavioural changes through knowledge improvement. The Health Belief Model (Rosenstock, 1966) suggests that preventive health behaviours are influenced, among other factors, by the perceived benefits of an action, beliefs regarding its effectiveness in reducing the threat of the disease, perceived barriers such as the potentially negative aspects of a particular action (pain, time loss etc.), and by cues to action such as reminders.

The screening program invitation letter provides information on screening procedure, on benefits associated with regular screening, and cues to action by reminding this targeted population about the availability of screening and the optimal frequency. Hence, providing this information may affect perceived benefits of screening and barriers (e.g., lower financial barriers by providing a free voucher for a mammogram), and these in turn might affect screening participation. Increased mammography take-up is associated with greater perceived susceptibility, lower barriers and higher perceived benefits of screening (Glanz et al., 2008).

Alternatively, in the transtheoretical model (Prochaska and Velicer, 1997) knowledge is key for people to move along the stages of behavioural changes: pre-contemplation, contemplation, preparation, action, maintenance and termination. For instance, from pre-contemplation to contemplation, an individual evolves from being unaware of the health behaviour to intend to start the behaviour and balance the pros and cons of such behaviour. Increases in knowledge resulting from information provision typically improve awareness and help the individual to make the trade-off of their decision. Therefore, information disseminated by breast cancer screening programs that increased knowledge could lead to mammogram use (Spencer et al., 2005).

Following the Grossman model (Grossman, 1972) and the inclusion of preventive care in the model of demand for health by Phelps (1978), health information is expected to have an impact on health behaviours because information improves the assessment of the marginal efficiency of health care consumption and prevention. If the marginal benefit of health prevention is higher than its marginal cost, the individuals will invest in their health. However, providing additional information may not have a homogeneous effect on knowledge and subsequent health care consumption. The efficiency in producing health is assumed to be influenced by the education level. The underlying mechanism, known as the allocative efficiency hypothesis, is that educated individuals are better able to internalise the new health information in their decision making process and to use it to improve their health through health care consumption.

There are important differences between these models, and they disregard other relevant dimensions such as social context. However, all three models - the Health Belief model, the Transtheoretical model and the Demand for health model - suggest that providing free information should increase health knowledge and may have an impact on subsequent health behaviours.

**Fig. 1:**
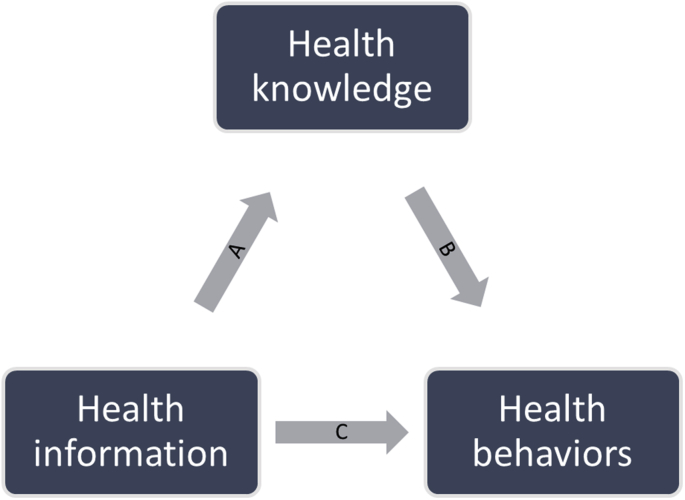
Relationship between health information, health knowledge and health behaviours. Notes: The figure depicts the conceptual model considered in this study.

Previous empirical research has focused on the effect of providing health information on health behaviour changes (Avery et al., 2007; Ippolito and Mathios, 1990, 1995; Pratt and Pratt, 1995), as represented by the arrow C in Fig. 1. Carrieri and Wuebker (2016) provide evidence of this effect in the case of breast cancer screening. They measure the effect of providing information on breast cancer screening through the invitation letter on mammography utilisation. Their identification strategy is similar to ours, i.e., they use variation in program implementation of breast cancer screening programs in Europe and variation in eligibility ages. They find a large positive effect, and this effect is greater among low educated women. A few studies have examined the effect of health knowledge levels on health behaviours as represented by the arrow B in Fig. 1, especially in the case of cigarette smoking (Jones and Kirigia, 1999; Kenkel, 1991) and doctor visits (Hsieh and Lin, 1997; Kenkel, 1990; Schmid, 2015). No study specifically documents the causal effect of providing health information on health knowledge.

The closest papers to ours are Ippolito and Mathios (1991), Hsieh et al. (1996) and Lillard and Önder (2019), which – to the best of our knowledge – are the only papers measuring the effects between health information, health knowledge and health behaviours. Ippolito and Mathios (1991) document the increase in knowledge about the relationship between fibre and cancer following the authorisation of producers to include health claims in their marketing strategy. This change in regulation also increased consumption of fibre cereals in the US. However, their before-after comparison does not allow a causal interpretation. Hsieh et al. (1996) examine the impact on smoking participation of health knowledge measured as the number of correct answers to questions about respondents' awareness of health consequences of smoking in Taiwan. They focus on the relationship between health knowledge and health behaviour, and use health information exposure (mass anti-smoking campaigns) as an instrumental variable to account for the endogeneity of the relationship between health knowledge and behaviours. They found that a 10% increase in health knowledge leads to a 4.8% reduction in smoking participation, and they observe a negative correlation between self-reported exposure to anti-smoking mass media campaigns (health information) and knowledge about smoking hazards. Lillard and Önder (2019) take advantage of time variation of anti-smoking article publications in the news to measure both the impact of informational flow and health knowledge stock on the probability to start or quit smoking in Turkey. While their article focuses on the impact of the informational flow on smoking behaviours, they control for health knowledge stock. Their results indicate that additional health information decreases smoking initiation for women and increases cessation for both men and women. In addition, health knowledge has a negative impact on initiation for women and a positive impact on cessation for both men and women. The correlation between health information flow and health knowledge stock is not reported.

Our paper adds to previous research in several ways. First, we provide evidence of the causal effect of health information on health knowledge. Hsieh et al. (1996) used a self-reported measure of exposure to anti-smoking campaigns whose effect on smoking is unlikely to be causal due to, e.g., cognitive dissonance. While Lillard and Önder (2019)'s health information provision variable is exogeneous, its relationship with health knowledge is not the primary focus of their paper. Second, none of those studies specifically track down the effect of information provision on behavioural changes going though variation in knowledge, while we implement a mediation analysis to precisely measure how changes in information provision affect behaviours via changes in health knowledge. Third, we explore whether the effect of information provision on knowledge and health behaviours differ by educational attainment, instead of assuming that better informed individuals are more educated. Lillard (2017) and Lillard and Önder (2019) examine how the effect of information provision or the effect of knowledge on the probability to quit smoking vary by educational attainment. We differ from theirs because we study the effect of information provision on knowledge by educational attainment. Fourth, we focus on breast cancer screening while the closest papers to ours studied smoking behaviours.

Our research question is especially relevant in the case of breast cancer screening. Breast cancer is the most frequently diagnosed cancer and the leading cause of death from cancer among women worldwide, accounting for one quarter of all new cancer cases and roughly 15% of cancer deaths (Torre et al., 2015). The existing literature suggests that women misperceive the risk of breast cancer and screening benefits (Barratt et al., 1997; Carman and Kooreman, 2014; Domenighetti et al., 2003; Gigerenzer et al., 2009), which raises the question of how information could improve individual's knowledge. Moreover, most developed countries have a breast cancer screening program inviting women to mammography screening. The invitation systematically provides information on breast cancer and the benefits of screening, yet its causal effect on knowledge is not known. Finally, from a methodological perspective, the gradual implementation of cancer screening programs in Europe provides an ideal setting to test the effect of health information on health knowledge.

In a public policy analysis perspective, our paper contributes to unravel the mechanisms at work in a large-scale health program. Considering that similar programs are implemented for colorectal and cervical screening throughout Europe and concerns over low participation in cancer screening (Altobelli et al., 2014; Altobelli and Lattanzi, 2015; Elfström et al., 2015), evidence on the effect of the different components of such programs seems essential to increase uptake. A related question was investigated by Offman et al. (2013) who conducted a randomized controlled trial on appointment time (office hours vs. out of office hours) to enhance accessibility to breast cancer screening in the UK.

## 2 Background on breast cancer screening

Mammography is the most important imaging procedure for breast cancer detection and diagnosis. The general aim is to enable early treatment of breast cancer, to improve survival rates and to reduce the need for aggressive treatments such as mastectomy. In all European countries, mammography carried out by a radiologist is available to all women upon prescription. A clinical exam is often performed with the mammography. While breast cancer screening can be covered by national health insurance, coverage varies by country. European breast cancer screening programs provide free mammography and a clinical exam to women, commonly aged 50 to 69, with no upfront fees. These programs typically also send an invitation letter to eligible women by mail every 2–3 years containing detailed information about the age range, screening interval and breast cancer screening procedure (which might differ between countries). The European guidelines (Perry et al., 2008) indicate (as a target for national programs) that at least 70% of invited women should attend breast cancer screening.

During the survey years considered in this paper (1997–1998), only the Netherlands, Luxembourg, Finland, the UK and Sweden had a national breast cancer screening program. The eligibility age range varies across countries (see Table A1 in the appendix). The minimum age window is 50–69, and the maximum age window is 40–74 (Altobelli and Lattanzi, 2014). Once eligible for these programs, women could receive a free mammography. Screening procedures were similar across countries with two views by mammography, double reading of negative mammograms, and 2 years screening intervals. Only the UK differed from the other countries as it had no double reading and a screening interval of 3 years. All screenings used screen-film mammograms because no digital mammograms were available at that time. The crucial element of the screening program for our empirical strategy is the letter of invitation conveying information to eligible women. Invitation letters for screening programs usually provide information about the potential benefits (and, less commonly, harms) of breast screening. A few studies have examined the informational content of some programs (Gummersbach et al., 2009; Jørgensen and Gøtzsche, 2004, 2006; Zapka et al., 2006). Unfortunately, no paper precisely documents the informational content of invitation letters of the screening programs in 1997/1998. Other papers provide evidence of similarities of the informational content of these programs in later years. Zapka et al. (2006) show that the Netherlands, Luxembourg, the UK and Finland had very similar informational content regarding the benefits of breast cancer screening. In addition, Finland and Luxembourg included information on the benefits of early treatment and manual breast examination (by a clinician and self-examination). For Sweden, the only available information is presented by Jørgensen and Gøtzsche (2006), whose study focuses on the benefits and harms of screening in terms of mortality. They find that Sweden had no information on those two aspects.

It's important to note that our information provision treatment, i.e., receiving an invitation letter for breast cancer screening, provides information to all eligible women. However, it is intended to lead to a doctor's appointment and subsequent mammography utilization, both of which could add an additional layer of information. In addition, women who are aware of their eligibility might obtain information through other media than the invitation letter. In all cases, we can expect an effect of information on knowledge triggered by program eligibility, although the change in knowledge is not solely due to the informational content of the invitation letter.

Furthermore, some eligible women may not receive the invitation. In the UK for instance, women will be invited for the first time within 3 years of their 50th birthday, such that it’s possible that they do not receive their invitation before age 53. A robustness check dropping women aged 50–52 shows that results are qualitatively similar. Another possibility might be that the postal mail fails to deliver it because addresses are not up to date. This is usually a very small share of the eligible population, and it should be accounted for by the country fixed effects and country-by-year fixed effects added in the robustness checks of our analysis.

## 3 Data and methods

### 3.1 Data

#### 3.1.1 Eurobarometer

The Eurobarometer surveys are regularly conducted as repeated cross-sectional surveys and include individuals in all current member states of the European Union. Topics are included to address current information needs of the European Commission and Parliament, and are therefore subject to frequent change. The Eurobarometer data are available for scientific use from the Eurobarometer Data Service at GESIS (GESIS, 2017). This study reports findings from a secondary data analysis of already existing data. Ethical approval for the study was therefore not deemed necessary.

For this study, we use data collected in April–June 1997 (EB47.2) and April–May 1998 (EB49), respectively. These are the only survey waves that asked respondents about their knowledge concerning breast cancer prevention and treatment, as well as women's use of preventive medical check-ups, e.g., mammography screening. The surveys were conducted in all 15 member states of the European Union at that time (Austria, Belgium, Denmark, Finland, France, Germany, Greece, Ireland, Italy, Luxembourg, Netherlands, Portugal, Spain, Sweden and the UK). We complement this dataset with information on breast cancer screening program availability from Altobelli and Lattanzi (2014).

The timing of the surveys is well suited for our study. We exploit variation in program existence between countries and eligibility across cohorts for causal identification. In addition, at the time of the survey there was a broad consensus about the benefits of mammography screening. Since the publication of Gøtzsche and Olsen (2000)'s meta-analysis of mammography screening, this consensus is increasingly questioned. The uncertainty surrounding the benefits and potential harms of breast cancer screening could have affected how information provided by the screening programs is perceived and taken into account. Our analysis does not suffer from this potential conflicting information, because the surveys were conducted before these developments.

#### 3.1.2 Outcomes: measuring health knowledge

In the Eurobarometer survey, women were asked for each of the following six statements whether they think the statement is true, false or whether they “don't know”.

- “The sooner a cancer is detected, the better it can be treated.”
- “A manual breast examination will detect signs of breast cancer.”
- “A mammography will detect signs of breast cancer.”
- “There are effective treatments for breast cancer.”
- “In most cases, you can be cured of breast cancer if it is detected early enough.”
- “Removal of the breast is the only way to be cured of breast cancer.”

Using these six items, we construct an index measuring the stock of health knowledge for each woman in line with Kenkel (1990)'s and Hsieh and Lin (1997)'s methods. We first assign a score to each answer for every statement described above, and then sum over all six items. We assign a score of 1 if women stated that the statement is “true”, and 0 if they responded “don't know” or “false”. The item on breast removal was reverse coded. The resulting index varies between 0 and 6. In a robustness check, we also explored an alternative scoring method, where we assign a value of −1 to “false” answers, 0 to “don't know” and a value of 1 to “true” answers. We also modelled all items individually as outcomes without assigning a score to the responses (see online appendix B).

As in previous studies on mammography use (Buchmueller and Goldzahl, 2018; Carrieri and Wuebker, 2016; Pletscher, 2017), breast cancer screening participation is based on self-reports referring to the past 12 months.

**Fig. 2:**
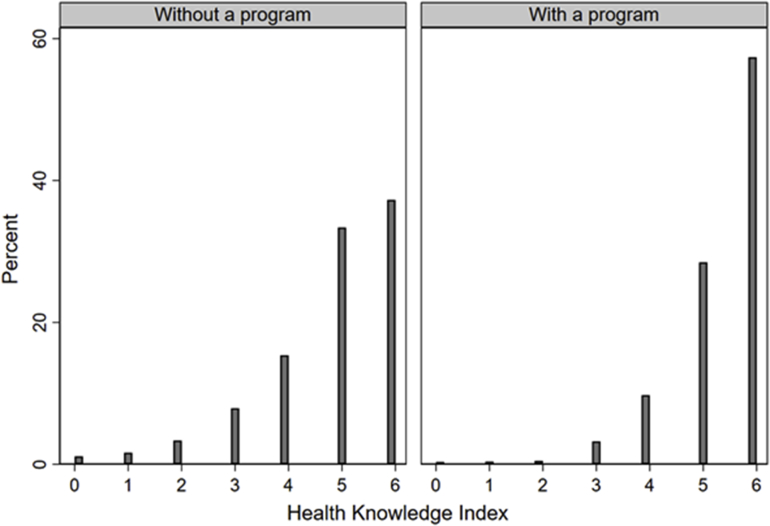
Distribution of the health knowledge index.

Source: Eurobarometer, own calculations. The figure shows the distribution of the health knowledge index in countries with and without a nationwide screening program.

Fig. 2 shows the distribution of the health knowledge index separately for women in countries with and without a screening program. We note that in both groups the distribution is heavily skewed to the left, with 85% of the sample scoring 4 or more points on the index in countries without a screening program (95% in countries with a screening program).

According to our health knowledge index, the level of knowledge is comparatively high, and higher than levels of knowledge reported in the existing literature. The explanation for this difference lies in the different level of difficulty of the questions used to construct the health knowledge index. Domenighetti et al. (2003) asked precise questions about the extent to which mammography reduces breast cancer deaths among women aged 50+ screened every 2 years for 10 years (with possible responses such as: it reduces mortality due to breast cancer by about a quarter, about a half, by about three-quarters, etc.), while the Eurobarometer presented broader and simpler statements, which could be answered with “true” or “false”. We note that for this paper we implicitly assume that women answer “don't know” due to a (perceived) lack of knowledge. However, it is possible that respondents might interpret these statements differently due to the ambiguous phrasing. For example, the statement “A mammography will detect signs of breast cancer.” could be understood as implying that a mammography will always detect breast cancer. Women who are aware of the possibility of false negative or false positive findings might therefore choose “don't know” as an answer. This would introduce measurement error in the health knowledge index, which would bias our results towards zero.

#### 3.1.3 Sample description

Our sample includes all women aged between 35 and 85, since breast cancer incidence is negligible below 35 and women older than 85 represent a highly selected group. Table 1 below provides summary statistics for our working sample. We note that health knowledge is relatively high overall. The health knowledge index shows an average of 5 out of 6. Yet, there is considerable heterogeneity between items. The table also shows considerable differences between women in countries with and without a screening program with respect to health knowledge and mammography use. However, women in countries without a screening program tend to be younger and finished education at a later age. Table A2 in the online appendix provides additional descriptive statistics for occupation, marital status and income.

**Table 1:** Summary statistics.

Variable	Mean	N	Difference by program	
*A. Health knowledge*				
*Health knowledge index*	4.965	10,610	0.545	***
*“The sooner a cancer is detected, the better it can be treated."*				
**True**	0.955	10,610	0.036	***
**False**	0.021	10,610	−0.018	***
**Don't Know**	0.023	10,610	−0.019	***
*“A manual breast examination will detect signs of breast cancer."*				
**True**	0.820	10,610	0.035	***
**False**	0.109	10,610	0.008	
**Don't Know**	0.071	10,610	−0.043	***
*“A mammography will detect signs of breast cancer."*				
**True**	0.916	10,610	0.033	***
**False**	0.034	10,610	0.002	
**Don't Know**	0.050	10,610	−0.036	***
*“There are effective treatments for breast cancer."*				
**True**	0.773	10,610	0.160	***
**False**	0.087	10,610	−0.062	***
**Don't Know**	0.140	10,610	−0.098	***
*“In most cases, you can be cured of breast cancer if it is detected early enough."*				
**True**	0.863	10,610	0.078	***
**False**	0.055	10,610	−0.024	***
**Don't Know**	0.081	10,610	−0.054	***
*“Removal of the breast is the only way to be cured of breast cancer."*				
**True**	0.200	10,610	−0.076	***
**False**	0.638	10,610	0.202	***
**Don't Know**	0.162	10,610	−0.126	***
*B. Other variables*				
*Mammography use in the past* 12 months	0.224	10,610	0.035	***
*Age*	54.134	10,610	−1.258	***
*Age when finished full-time education*				
**Before age 16**	0.415	10,610	−0.145	***
**Age 16**–**19**	0.371	10,610	0.036	***
**Age 20 and above**	0.204	10,610	0.099	***
**Still studying**	0.009	10,610	0.010	***
*Living in country with a screening program*	0.305	10,610		
*Living in country with a program and within age range (treated obs.)*	0.141	10,610		

### 3.2 Methods

#### 3.2.1 Difference-in-differences estimation

Eligibility for screening in a national screening program is not randomly distributed. Table 1 in the manuscript demonstrates that there are systematic differences in knowledge and screening between women in countries with a program and those in countries without a screening program. Similarly, eligibility for screening in an organized program is restricted to a certain age range, and therefore we would expect systematic differences between eligible and ineligible women in countries with a program, e.g., due to cohort differences in education. Such differences between countries as well as between age cohorts can bias our estimates, and we therefore apply a DID design. Intuitively, we compare the difference in health knowledge between eligible (inside the age range) and ineligible women (outside the age range) in countries with a screening program (first difference) to the difference in health knowledge between women in the same age groups in countries without a screening program (second difference). Crucially, the eligibility age range varies across countries (e.g., in Sweden the lower eligibility age is 40 compared to 50 in the UK), and we therefore do not only rely on age differences. This setup deviates slightly from the common DID approach, where treatment assignment varies across regions and over time. However, DID designs can be applied more broadly (Wing et al., 2018), and our modelling approach closely follows an earlier study on the effects of health information on screening participation (Carrieri and Wuebker, 2016).

The DID approach assumes that differences in (observed and unobserved) confounders only affect the level of health knowledge, but that in the absence of treatment the trends in countries with and without a screening program would be the same. This assumption is referred to as the “common trends assumption” (CTA). For example, Table 1 shows that there is a difference in education between women in countries with and without a screening program, which likely explains some of the difference in health knowledge. The CTA implies that (in the absence of screening programs) the trends in health knowledge would have been similar in all countries, for instance because educational expansion has led to higher educational attainment among younger women across all countries.

The CTA cannot be formally tested, because it refers to counterfactual outcomes. However, we argue that this assumption is likely to hold for four reasons: First, the European Union made uniform recommendations on breast cancer screening program guidelines across countries, and following these recommendations all countries except Greece had implemented an organized program by 2010. Second, the epidemiological literature suggests that age-specific incidence and mortality rates for breast cancer do not differ meaningfully across countries (Althuis et al., 2005; Bray et al., 2004). Third, we provide visual evidence for this assumption.

**Fig. 3:**
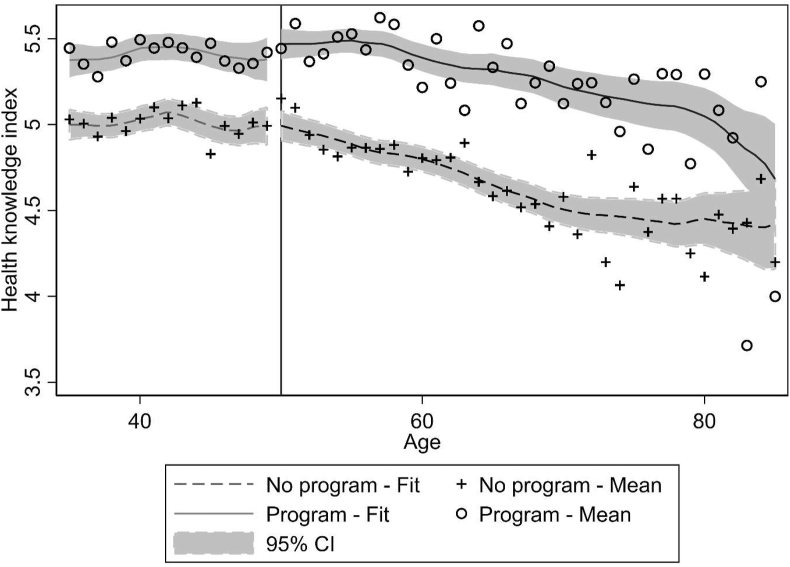
Trends in health knowledge by age.

Source: Eurobarometer, own calculations. The fitted lines show local mean fits of mammography use in the past 12 months against age. The vertical line marks age 50, the most common lower limit for screening program eligibility in our sample. It should be noted that some countries offer screening from age 40 or 45 onwards, see Table A1 in the online appendix.

Fig. 3 shows levels of health knowledge by age in countries with and without a screening program. Until age 50 (the lower age limit for screening program eligibility in most countries), levels of health knowledge in both groups exhibit little variation with age and the trends appear broadly parallel, which we interpret as support for the CTA. From age 50 onwards, the trends seem to diverge. While in countries with a screening program levels of health knowledge remain stable until about age 60 and decline thereafter, average health knowledge in countries without programs seems to decline earlier from around age 50. This decline in health knowledge could either reflect cohort trends, e.g., higher levels of education among younger women, or age-related shifts in health care consumptions such as a decline in gynaecologist visits (see Figure C1 in the online appendix). Taken together, this suggests that screening program eligibility positively affects health knowledge by (partially) offsetting the decline observed in countries without a program. We also conduct a placebo check and examine trends in the retirement probability by age. Intuitively, if trends in unrelated variables are not parallel in countries with and without a screening program, this would raise doubts about the validity of the CTA. In contrast, parallel trends suggest that the covariate can be considered balanced between the treatment and control group. Figure C2 in the online appendix shows very similar patterns in countries with and without screening programs.

Fourth, we can also test for age-specific differences among women below the eligibility age (see Table 3 in section 4.1). Overall, we conclude that the CTA is likely to hold.

**Table 2:** DID estimation - health knowledge index.

	Main model	Additional covariates	Country-by-year FE	Cubic age trend	5-year age groups	No age controls	Without older control group	Alternative Scoring Method	Without women in the first interval
**Program**	0.485***	0.467***	0.601***	0.479***	0.476***	0.469***	0.429***	0.637***	0.485***
	(0.073)	(0.075)	(0.107)	(0.073)	(0.073)	(0.073)	(0.071)	(0.093)	(0.075)
**Age range**	−0.025	−0.028	−0.025	−0.017	0.019	0.007	−0.016	−0.057	−0.046
	(0.042)	(0.043)	(0.042)	(0.043)	(0.057)	(0.033)	(0.048)	(0.055)	(0.045)
**Program x Age range**	0.144***	0.149***	0.145***	0.147***	0.146***	0.121**	0.214***	0.174***	0.171***
	(0.047)	(0.048)	(0.047)	(0.047)	(0.047)	(0.049)	(0.047)	(0.062)	(0.048)
*N*	*10,610*	*10,603*	*10,610*	*10,610*	*10,610*	*10,610*	*9006*	*10,610*	*10,023*

Source: Eurobarometer, own calculations. The estimates come from a linear model controlling for a quadratic age trend, education in four categories, country- and year-fixed effects. Standard errors clustered by country and age in parentheses. The additional covariates in column 2 are occupation, marital status, household income in country-specific quartiles (incl. a category for missing values), whether children under 15 are present in the household and whether other persons were present during the interview (see Table A2 in the online appendix). Column 7 excludes women below the upper age limit for screening eligibility. In column 8, the health knowledge index is assigned 1 point for a “true” answer, 0 points for “don't know” and −1 point for “false” answers. *p < 0.1, **p < 0.05, ***p < 0.01.

**Table 3:** Heterogeneity by age.

Dependent variable: Health knowledge index				
**Age group**	*% eligible in treatment group*	**Coefficient**	**Standard Error**	
35–39	0	−0.047	0.075	
40–44	20	−0.078	0.076	
45–49	17	*Reference group*		
50–54	100	0.078	0.087	
55–59	100	0.165	0.081	**
60–64	100	0.130	0.088	
65–69	93	0.282	0.093	***
70–74	43	0.223	0.113	**
75–79	0	0.164	0.139	
80–85	0	0.234	0.166	

Source: Eurobarometer, own calculations. The estimates come from a linear regression model. The coefficient estimates in column 3 show the estimated interaction effects between the program indicator and the specified age groups. Control variables include dummy variables for 5-year age groups, four education categories, country- and year-fixed effects. Standard errors are clustered on the country-age level. Significance: *p < 0.1, **p < 0.05, ***p < 0.01.

We estimate the following linear regression model using ordinary least squares:

(1) $$knowledge_{i,c,t}=\beta_{0}+\beta_{1}program_{c,t}+\beta_{2}agerange_{i,c,t}+\beta_{3}program_{c,t}*agerange_{i,c,t}+\beta_{4}age_{i,t}+\beta_{5}age_{i,t}^{2}+\beta_{6}educ_{i,t}+\gamma_{c}+\delta_{t}+\varepsilon_{i,t\;}$$

Here, *i* denotes the individual, *c* the country and *t* the year. $program_{c,t}$ is a binary indicator for observations from countries with a screening program. $agerange_{i,c,t}$ is a binary indicator for women within the age range of their country's (current or future) screening program. $program_{c,t}*agerange_{i,c,t}$ is an interaction effect, essentially denoting eligible women within countries with a screening program. The reference category are women living in countries without a current screening program, and which are outside of the age range of their future national program. $\beta_{3}$ measures the treatment effect. We include a quadratic trend for age, four categories of education, country- and year-fixed effects as control variables in all models. These fixed effects should control for most of the variation in health knowledge across countries. The education categories are defined as - “finished fulltime education before age 16”, “finished fulltime education between the age of 16 and 19”, “finished fulltime education after age 19” and “still studying”. The first three categories should broadly correspond to a low, medium and high level of education. Standard errors are clustered by country and age.

It should be noted that the treatment effect identified by equation (1) is an Intention-To-Treat (ITT) effect, since we observe screening program eligibility rather than the actual receipt of an invitation to screen.

#### 3.2.2 Mediation analysis

If information provided by breast cancer screening programs affects women's health knowledge, then this raises the question whether these changes in health knowledge affect screening participation. The increase in mammography use following the introduction of breast cancer screening programs is likely driven by several mechanisms, such as a reduction in the cost of screening or reminder effects or the provision of health information. We argue that the provision of health information should affect screening participation only through its effect on health knowledge, whereas the other mechanisms should have no effect on health knowledge. Thus, we can disentangle the effect of health information on screening participation from other mechanisms by conducting a mediation analysis with health knowledge as the mediator.

We conduct a causal mediation analysis, following an approach developed by Imai et al. (Imai et al, 2010a, 2010b, 2013) and implemented by Hicks and Tingley (2011). We estimate a linear model regressing the health knowledge index on program eligibility and control variables using the same DID model described above (mediator model). Then, we estimate a probit model regressing mammography use on program eligibility and the control variables in our DID model as well as the health knowledge index (outcome model). In this model, screening program eligibility primarily captures the mechanisms that do not operate through health knowledge, because health knowledge is controlled for in the regression. Based on these regressions, the algorithm developed by Hicks and Tingley (2011) calculates the mediation effects using simulations drawn from the estimated distributions of the model parameters. Intuitively, the effect of information on screening program participation (“mediation effect”) is obtained from the mediator model of screening program eligibility on knowledge as well as the effect of health knowledge on screening participation in the outcome model, whereas all other mechanisms (“direct effect”) are captured by the estimated effect of screening program eligibility on screening participation in the outcome model. We are interested in the average direct effect, the average mediation effect and the % of the total effect mediated. The “% of the total effect mediated” can be interpreted as a measure of the relative importance of health knowledge for the relationship between screening program eligibility and mammography use.

Causal mediation analysis requires the assumption of “sequential ignorability” (Imai et al., 2010b). This means that *(i)* treatment assignment is independent of the outcome and the mediator conditional on pre-treatment covariates, and *(ii)* the mediator is independent of the outcome conditional on treatment assignment and pre-treatment covariates (Imai et al, 2010b, 2013). While this assumption is not testable, the algorithm by Hicks and Tingley (2011) allows us to assess the sensitivity of our results to violations of this assumption by recalculating the mediation effects for all possible values of a sensitivity parameter ρ, which expresses the correlation between the error terms in the models for the mediator and the outcome.

## 4 Results

### 4.1 The effect of information provision on health knowledge

Table 2 shows information provision as part of an organised screening program increases health knowledge by 0.14 points on the health knowledge index, which corresponds to a 2.9% increase based on the mean reported in Table 1. This effect is robust to several changes of the model specification, e.g., controlling for additional demographic characteristics (occupation, marital status, household income, whether children under 15 are present in the household as well as whether additional persons were present during the interview). We also assessed the robustness to controlling for country-by-year fixed effects, a cubic age polynomial, dummies for 5-year age groups, not controlling for age, an alternative scoring method which decreases the health knowledge index by one point for “false” answers, and excluding the older control group or women within the first screening interval.

We also examine the health knowledge statements separately to determine whether the increase in health knowledge is driven by specific items (see online appendix B). We find that the positive effect estimated for the health knowledge index seems to be primarily driven by the statement on treatments (“There are effective treatments for breast cancer”). We also check that our results are robust to excluding specific treated countries (see Table C1 in the online appendix). Finally, France, Denmark, Spain, Italy and Portugal had regional breast cancer screening programs at the time of the survey. Those programs operate similar to nationwide programs, but only in a specific region. We replicated our analysis using regional rather than nationwide programs (available in online appendix D). The results are qualitatively similar.

Finally, we examine heterogeneity across age groups by estimating our DID model with an interaction term between program existence and five-year age groups (rather than the age range). The results in Table 3 show the estimated difference between countries with and without a screening program in the specified age group *relative* to the difference in the age group 45–49. This also allows us to formally test for differences in trends of health knowledge in ineligible (i.e., pre-treatment) age groups. We see that for the age groups 35–39 and 40–44 (in which only Swedish women were eligible for screening), the difference between countries with and without a screening program does not significantly differ from the difference in the 45–49 age group. This further suggests that the CTA is likely to hold. Moreover, we see that the effect of health information on health knowledge tends to be stronger among older age groups.

### 4.2 The effect of screening program eligibility on mammography use

Next, we estimate our DID model to examine whether screening program eligibility affects screening participation. Figure C3 in the online appendix shows trends in mammography use by age. Below age 50 the trends in both groups of countries appear to be parallel. At age 50, we observe a stark increase in mammography use in countries with a screening program. Moreover, the trends diverge between age 50 and age 75, indicating that screening program eligibility increases rates of mammography use.

Table 4 shows that screening program eligibility increases the probability to have had a mammography in the past 12 months by 20.1 percentage points, which is comparable in magnitude to the effect found in a previous study using European data (Carrieri and Wuebker, 2016). Moreover, this effect is also highly robust to the specification changes outlined above.

**Table 4:** DID estimation - mammography use.

	Main model	Additional covariates	Country-by-year FE	Cubic age trend	5-year age groups	No age controls	Without older control group	Without women in the first interval
**Program**	−0.158***	−0.153***	−0.142***	−0.164***	−0.161***	−0.166***	−0.180***	−0.161***
	(0.029)	(0.029)	(0.038)	(0.029)	(0.028)	(0.031)	(0.033)	(0.030)
**Age range**	0.006	0.006	0.005	0.014	−0.042	0.083***	0.011	0.004
	(0.014)	(0.014)	(0.014)	(0.014)	(0.025)	(0.011)	(0.020)	(0.015)
**Program x Age range**	0.201***	0.198***	0.201***	0.205***	0.207***	0.199***	0.227***	0.209***
	(0.022)	(0.021)	(0.022)	(0.021)	(0.021)	(0.023)	(0.022)	(0.023)

*N*	*10,610*	*10,603*	*10,610*	*10,610*	*10,610*	*10,610*	*9006*	*10,023*

Source: Eurobarometer, own calculations. The estimates come from a linear model controlling for a quadratic age trend, education in four categories, country- and year-fixed effects. Standard errors clustered by country and age in parentheses. The additional covariates in column 2 are occupation, marital status, household income in country-specific quartiles (incl. a category for missing values), whether children under 15 are present in the household and whether other persons were present during the interview (see Table A2 in the online appendix). Column 7 excludes women below the upper age limit for screening eligibility. *p < 0.1, **p < 0.05, ***p < 0.01.

### 4.3 Mediation analysis

Table 5 shows the estimated mediation effects. The average mediation effect, i.e., the effect of screening program eligibility on mammography use operating through changes in health knowledge, is estimated to be about 0.5 percentage points, or 2.4% of the total effect of screening program eligibility on mammography use. While the confidence interval suggests that the average mediation effect is significantly different from zero, it is nevertheless small and contributes little to the total effect of screening program eligibility on mammography use. This is partly due to the limited effect of health knowledge on mammography use – the regression results suggest that a 1-point increase in the health knowledge index only increases the probability of having a mammography by 3.1 percentage points.

**Table 5:** Mediation analysis.

	Mean	S.E.	90% CI	
*A. Regression results*				
Effect of Eligibility on Knowledge	0.144	0.047	[0.066, 0.222]	***
Effect of Eligibility on Screening Participation	0.182	0.020	[0.149, 0.214]	***
Effect of Knowledge on Screening Participation	0.031	0.004	[0.025, 0.037]	***
*B. Mediation effects*				
Average Mediation	0.005		[0.002, 0.008]	
Average Direct Effect	0.208		[0.168, 0.250]	
% of Total Effect mediated	0.024		[0.020, 0.029]	

Source: Eurobarometer, own calculations based on Hicks and Tingley (2011). Panel A shows estimated marginal effects from the regression models. All models include controls for a quadratic age trend, four levels of education, country- and year-fixed effects. Standard errors are clustered on country- and age-level. Significance: *p < 0.1, **p < 0.05, ***p < 0.01.

The mediation analysis assumes “sequential ignorability”. Violations of this assumptions can be expressed by the sensitivity parameter ρ, which captures the correlation between the errors in the outcome and the mediator models. In the present case, the sequential ignorability assumption might not hold for several reasons. First, mammography use is reported retrospectively, and thus it is possible that our estimates are affected by reverse causality. Individuals might also change their health information based on their screening activity to avoid cognitive dissonance. In both cases, this would imply a positive value for ρ,i.e., women with better health knowledge are more likely to screen. Second, fear and anticipated emotions from undertaking breast cancer screening due to expecting bad news might discourage women with better knowledge on breast cancer screening and treatment from screening (Bousquet, 2016; Kőszegi, 2003). This would imply a negative value for ρ.

**Fig. 4:**
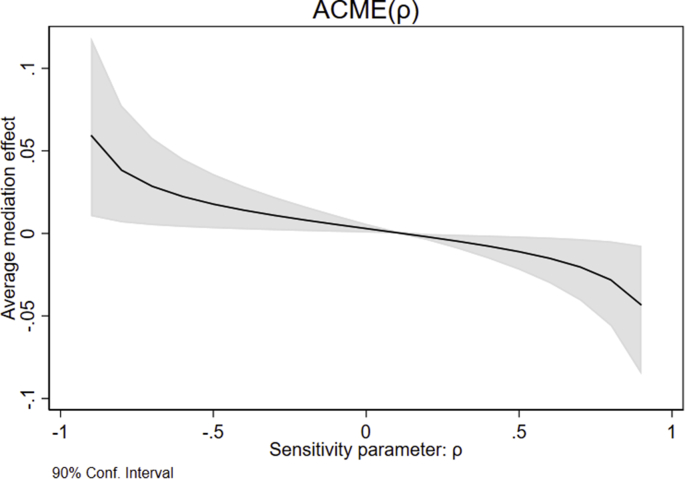
Sensitivity analysis for the average causal mediation effect (ACME).

Source: Eurobarometer, own calculations based on Hicks and Tingley (2011). The figure shows the average causal mediation effect (ACME) as a function of a sensitivity parameter, which expresses the correlation between the error terms in the regression model for the mediator and the regression model for the outcome. The figure is produced by estimating the average mediation effect for different assumed values of the sensitivity parameter using the simulation-based algorithm developed by Hicks and Tingley (2011).

Fig. 4 plots the average causal mediation effect (ACME) against the value of ρ. We note that even for extreme values of ρ the average mediation effect is only estimated to be about 5 percentage points. For more realistic values of ρ between −0.5 and + 0.5, the curve is relatively flat, implying ACMEs of 2 percentage points or less. This suggests that, even with a moderate degree of endogeneity, health knowledge mediates less than 10% of the total effect of screening program eligibility on mammography use. Thus, we conclude that the mediation effect is unlikely to be of practical relevance.

### 4.4 Heterogeneity by education

It seems plausible that the effect of information provision differs by education levels. Better educated women are likely to have a higher stock of health knowledge, and therefore the information provided as part of the screening program might not be new to them. In contrast, education is also often considered as a proxy variable for cognitive skills, which would imply that better educated women are also more likely to process new information efficiently. Table 6 shows estimates from a mediation analysis for each education group separately.

**Table 6:** Mediation analysis by education.

	Low education (n = 4407)		Medium education (n = 3936)		High education (n = 2167)		
	Mean	90% CI	Mean	90% CI	Mean		90% CI
*A. Regression Results*							
Effect of Eligibility on Knowledge	0.153*	[0.019, 0.288]	0.098	[-0.022, 0.219]	0.059		[-0.078, 0.197]
Effect of Eligibility on Screening Participation	0.149***	[0.094, 0.204]	0.217***	[0.168, 0.267]	0.130***		[0.070, 0.190]
Effect of Knowledge on Screening Participation	0.037***	[0.028, 0.045]	0.029***	[0.019, 0.039]	0.014		[-0.001, 0.028]
*B. Mediation Effects*							
Average Mediation	0.006	[0.001, 0.012]	0.003	[-0.001, 0.007]		0.001	[-0.001, 0.004]
Average Direct Effect	0.170	[0.102,0.245]	0.250	[0.189, 0.316]		0.143	[0.073, 0.221]
% of Total Effect mediated	0.035	[0.025, 0.057]	0.012	[0.010, 0.016]		0.006	[0.004, 0.011]

These results indicate a clear gradient: among low educated women, a 1-point increase in health knowledge increases the probability of mammography use by 3.7 percentage points, compared to 2.9 percentage points in the medium education group and 1.4 percentage points in the high education group. The effect of screening program eligibility on mammography use also differs by education. Taken together, Table 6 shows that the relative importance of health knowledge as a mediator decreases with increasing education. Yet, even among low educated women the mediation effect is small.

## 5 Discussion

This paper shows that women who become eligible to screen in their national breast cancer screening program have better knowledge of breast cancer prevention and treatment. This effect is particularly driven by an increase in knowledge about treatment.

While we find a significant effect of information provision on health knowledge, a mediation analysis suggests that it is unlikely that the change in health knowledge leads to substantial changes in health behaviour. In line with earlier studies, we find that screening program eligibility has a large effect on mammography use, however, most of the increase in mammography use can be attributed to the direct effect of the screening program, while only about 2.4% of the total effect operate through changes in health knowledge. Finally, we examine heterogeneity by education. The mediation analysis shows a clear gradient in the average mediation effect across education groups, yet even among low educated women the mediation effect is small.

In other words, it implies that for breast cancer screening programs almost the entire change in behaviours can be attributed to factors other than health knowledge e.g., the reduction in access barriers (including costs) or behavioural effects (e.g., reminders). It's also possible that the direct effect includes changes in knowledge that are not captured by our health knowledge indicator.

Using survey data from 1997 to 1998, an obvious concern is the extent to which our results are still relevant nowadays. We argue that the most important changes since 1998 concern screening uptake and the type of information provided in the invitation letters.

Assuming that neither of those two features have changed in the past two decades, the health knowledge distribution should not be considerably different since it was already high and concentrated around the mean in the late 90s. Consequently, our results would be similar. In contrast, if screening uptake and information contents changed, we can speculate how these changes might affect our conclusions.

First, screening take up in European countries has increased in the past two decades, and reaches more than 75% in four EU member states (Eurostat, 2018). Higher rates imply that the scope for information provision by mail to affect take-up is reduced. The population of eligible women that remains unscreened has particular characteristics such as disabilities or no access to mammography facilities. Providing more information to this population is unlikely to be enough to change their behaviour.

Second, the information provided in the invitation letter during the late 90s aimed at convincing eligible women to take up mammography, by emphasizing the benefits of participating in a breast cancer screening program. More than a decade later, an institutional shift – induced by the alarming evidence on overdiagnosis and overtreatment – occurred in the communication strategy adopted by breast cancer screening programs. They advocate that eligible women should take an informed decision based on balanced information mentioning the downsides of screening, and not only the benefits. This new type of information may have an impact on health knowledge and subsequent mammography utilization. Future research on this topic would improve our understanding of the consequences of this change in informational content.

## Credit statement

**Peter Eibich:** Conceptualization, Methodology, Validation, Formal Analysis, Data Curation, Writing – Original Draft, Writing – Review & Editing, Visualization. **Léontine Goldzahl:** Conceptualization, Methodology, Writing – Original Draft, Writing – Review & Editing.

## Funding

Peter Eibich acknowledges generous support by the 10.13039/100010269Wellcome Trust (Society & Ethics Research Fellowship 203208/Z/16/Z).

## Declaration of competing interest

The authors declare no conflicts of interest.
