# Supplementary material for: Health information provision, health knowledge and health behaviours: Evidence from breast cancer screening
Source: Soc Sci Med. 2020 Nov;265:113505. doi: 10.1016/j.socscimed.2020.113505 (PMC7768188; doi:10.1016/j.socscimed.2020.113505)
Supplement: Multimedia component 1 [file mmc1.docx]

**Online Appendix**

Contents

[A. Additional tables 2](#_Toc44511284)

[B. Multinomial Logit Model 4](#_Toc44511285)

[C. Robustness Checks 6](#_Toc44511286)

[Figure C.1 Trends in gynaecologist visits by age 6](#_Toc44511287)

[Figure C.2 Trends in retirement by age 7](#_Toc44511288)

[Figure C.3 Trends in mammography use by age 8](#_Toc44511289)

[Table C.1: Excluding countries from the treatment group 9](#_Toc44511290)

[D. Regional-level Analysis 10](#_Toc44511291)

[References for the Appendix 12](#_Toc44511292)

# Additional tables

| **Table A.1: Breast cancer Screening program characteristics** | | |
| --- | --- | --- |
|  |  |  |
|  | *Year of implementation (nationwide coverage)* | *Age range* |
|  |  |  |
| Finland | 1989 | 50-69 |
| Luxembourg | 1992 | 50-69 |
| UK | 1995 | 50-70^a^ |
| Sweden | 1996 | 40-74 |
| Netherlands | 1997 | 50-74^b^ |
| Belgium | 2001 | 50-69 |
| France | 2004 | 50-74 |
| Portugal | 2005 | 45-69^c^ |
| Italy | 2007 | 50-69 |
| Austria | 2008 | 40-69 |
| Ireland | 2008 | 50-64 |
| Germany | 2009 | 50-69 |
| Spain | 2009 | 50-69^d^ |
| Denmark | 2010 | 50-69 |
| Greece | None | - |
| Source: Altobelli and Lattanzi (2014). | |  |
| ^a^. In the dataset we distinguish between Great Britain and Northern Ireland. In Northern Ireland, the age range of the program was 50-64 until 2003. | | |
| ^b^. The age range was 50-70 in 1997, and 50-74 from 1998 onwards. | | |
| ^c^. The age range is 50-69 in the Algarve region. | | |
| ^d^. The age range is 45-69 in 5 out of 17 NUTS-II regions. | | |

| **TableA.2 : Summary statistics of additional covariates** | | | | | |  |
| --- | --- | --- | --- | --- | --- | --- |
| **Variable** | | **Mean** | **N** | **Difference by program** | |  |
| *Occupation* |  |  |  |  |  |  |
|  | Homemaker | 0.272 | 10603 | -0.059 | *** |  |
|  | Student | 0.009 | 10603 | 0.008 | *** |  |
|  | Unemployed | 0.051 | 10603 | -0.005 |  |  |
|  | Retired or permanently sick | 0.290 | 10603 | -0.026 | *** |  |
|  | Farmer | 0.011 | 10603 | -0.006 | *** |  |
|  | Professional | 0.009 | 10603 | 0.003 |  |  |
|  | Shop owner, craftsmen, other self employed | 0.033 | 10603 | -0.023 | *** |  |
|  | Business proprietor, owner of a company | 0.010 | 10603 | 0.004 | * |  |
|  | Employed professional | 0.009 | 10603 | 0.012 | *** |  |
|  | General management | 0.007 | 10603 | 0.000 |  |  |
|  | Middle management | 0.055 | 10603 | 0.020 | *** |  |
|  | Employed, working at a desk | 0.073 | 10603 | 0.016 | *** |  |
|  | Employed, travelling | 0.013 | 10603 | -0.002 |  |  |
|  | Employed, service job | 0.077 | 10603 | 0.052 | *** |  |
|  | Supervisor | 0.004 | 10603 | 0.002 |  |  |
|  | Skilled manual worker | 0.032 | 10603 | -0.004 |  |  |
|  | Unskilled manual worker | 0.044 | 10603 | 0.009 | * |  |
| *Marital status* |  |  |  |  |  |  |
|  | Single | 0.085 | 10603 | 0.010 |  |  |
|  | Married | 0.595 | 10603 | -0.024 | ** |  |
|  | Living as married | 0.036 | 10603 | 0.027 | *** |  |
|  | Separated | 0.079 | 10603 | 0.032 | *** |  |
|  | Divorced | 0.017 | 10603 | 0.000 |  |  |
|  | Widowed | 0.188 | 10603 | -0.045 | *** |  |
| *Children under 15 present in the household* | | 0.277 | 10603 | 0.057 | *** |  |
| *Other persons present during the interview* | | 0.836 | 10603 | 0.027 | *** |  |
| *Income (country-specific quartiles)* | |  |  |  |  |  |
|  | 1st quartile | 0.241 | 10603 | 0.004 |  |  |
|  | 2nd quartile | 0.179 | 10603 | 0.019 | ** |  |
|  | 3rd quartile | 0.170 | 10603 | 0.013 | * |  |
|  | 4th quartile | 0.154 | 10603 | 0.036 | *** |  |
|  | Missing | 0.256 | 10603 | -0.072 | *** |  |
| Source: Eurobarometer, own calculations. Column 5 shows the estimated difference between observations in countries with and without an organized screening programs. P-values are based on a two-sided t-Test with unequal variances. Significance: *** p<0.01; ** p<0.05; * p<0.1. | | | | | |  |
|  |  |  |  |  |  |  |
|  |  |  |  |  |  |  |
|  |  |  |  |  |  |  |

# Multinomial Logit Model

When analysing the six health knowledge items individually, we estimate our difference-in-differences design with a multinomial logit regression model to allow for the possibility that information provision has different effects on the likelihood to answer “true”, “false” and “don’t know”. For example, women who answer “don’t know” might be more likely to update their health knowledge when receiving new information, while women answering “false” might already have prior information from own experiences or their social networks. The results are shown in Table B.1 below.

| **Table B.1: Marginal effects from a multinomial logit regression** | | | | | | | | | | | | | | | | | | |  |
| --- | --- | --- | --- | --- | --- | --- | --- | --- | --- | --- | --- | --- | --- | --- | --- | --- | --- | --- | --- |
| **Answer \ Outcome** | **The sooner a cancer is detected, the better it can be treated.** | |  | **A manual breast examination will detect signs of breast cancer.** | |  | **A mammography will detect signs of breast cancer.** | |  | **There are effective treatments for breast cancer.** | |  | **In most cases, you can be cured of breast cancer if it is detected early enough.** | |  | **Removal of the breast is the only way to be cured of breast cancer.** | |  |  |
| **True** | -0.002 |  |  | 0.011 |  |  | 0.022 | * |  | 0.058 | *** |  | 0.007 |  |  | -0.001 |  |  |  |
|  | (0.006) |  |  | (0.014) |  |  | (0.013) |  |  | (0.021) |  |  | (0.015) |  |  | (0.016) |  |  |  |
| **False** | 0.005 |  |  | -0.003 |  |  | -0.015 |  |  | -0.017 |  |  | -0.003 |  |  | 0.029 |  |  |  |
|  | (0.018) |  |  | (0.011) |  |  | (0.014) |  |  | (0.014) |  |  | (0.010) |  |  | (0.018) |  |  |  |
| **Don't know** | -0.003 |  |  | -0.009 |  |  | -0.007 |  |  | -0.041 | ** |  | -0.005 |  |  | -0.028 | * |  |  |
|  | (0.019) |  |  | (0.011) |  |  | (0.010) |  |  | (0.017) |  |  | (0.012) |  |  | (0.015) |  |  |  |
|  |  |  |  |  |  |  |  |  |  |  |  |  |  |  |  |  |  |  |  |
| Average % True | 0.955 |  |  | 0.820 |  |  | 0.916 |  |  | 0.773 |  |  | 0.863 |  |  | 0.200 |  |  |  |
| N | *10,610* |  |  | *10,610* |  |  | *10,610* |  |  | *10,610* |  |  | *10,610* |  |  | *10,610* |  |  |  |
| Sources: Eurobarometer, own calculations. The coefficients are marginal effects of screening program eligibility from a multinomial logistic regression model. All models include controls for education, a quadratic age trend, country- and year-fixed effects. All models include observations aged 35 and above. Standard errors are clustered on country- and age-level. Significance: * p<0.1; ** p<0.05; *** p<0.01. | | | | | | | | | | | | | | | | | | |  |
|  |  |  |  |  |  |  |  |  |  |  |  |  |  |  |  |  |  |  |  |
|  |  |  |  |  |  |  |  |  |  |  |  |  |  |  |  |  |  |  |  |

# Robustness Checks

## Figure C.1 Trends in gynaecologist visits by age


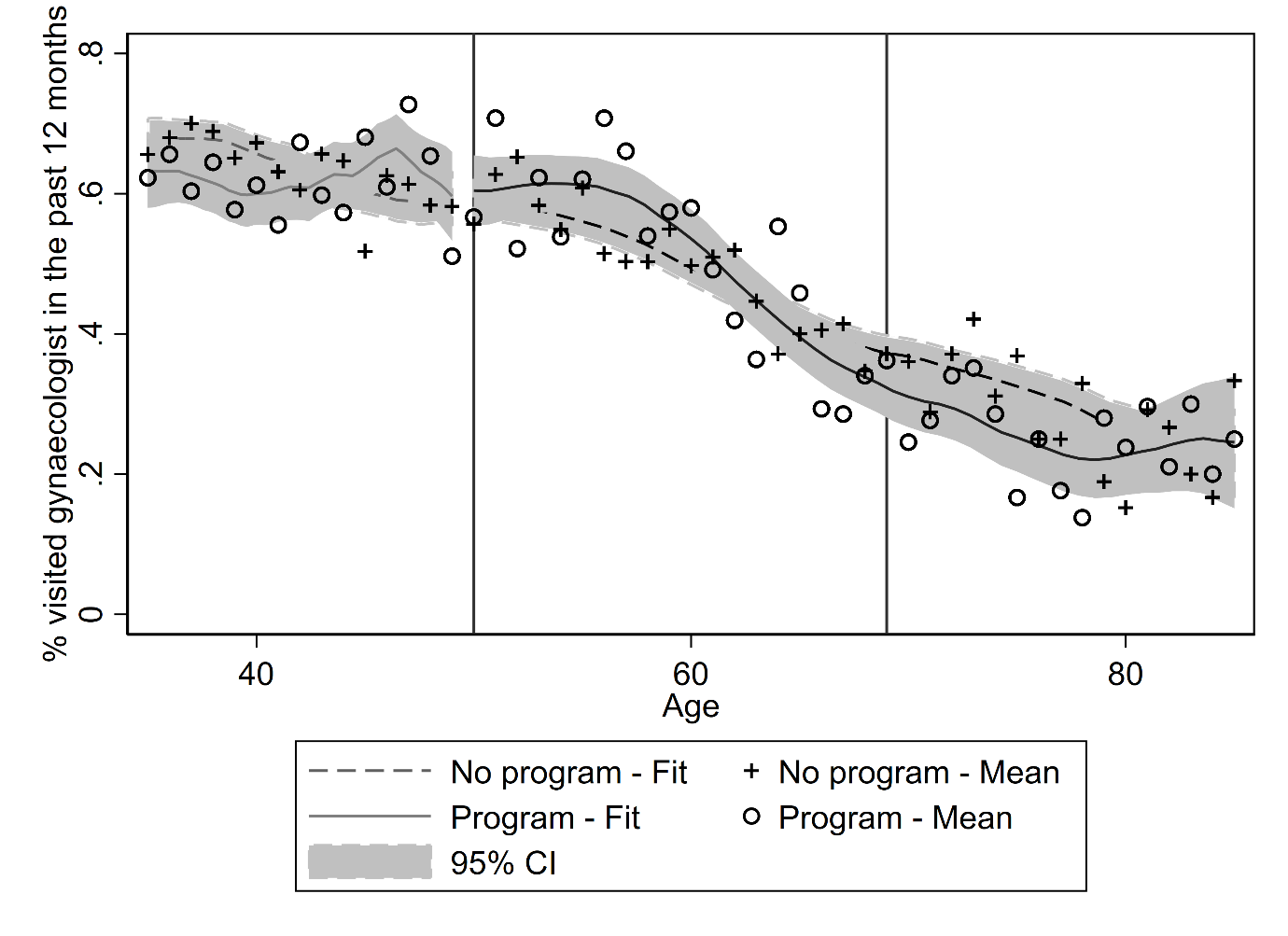


Source: Eurobarometer, own calculations. The fitted lines show local mean fits of gynaecologist visits in the past 12 months against age. Gynaecologist visits are measured by a binary indicator, which takes on the value of 1 if a woman reports having had a manual breast examination, a pap smear test, an examination of the ovaries, an osteoporosis test or any other gynaecological examination in the past12 months. The vertical lines mark ages 50 and 69, the most common lower and upper limit for screening program eligibility in our sample. It should be noted that some countries offer screening from age 40 or 45 onwards, see Table A.1 in the online appendix.

## Figure C.2 Trends in retirement by age


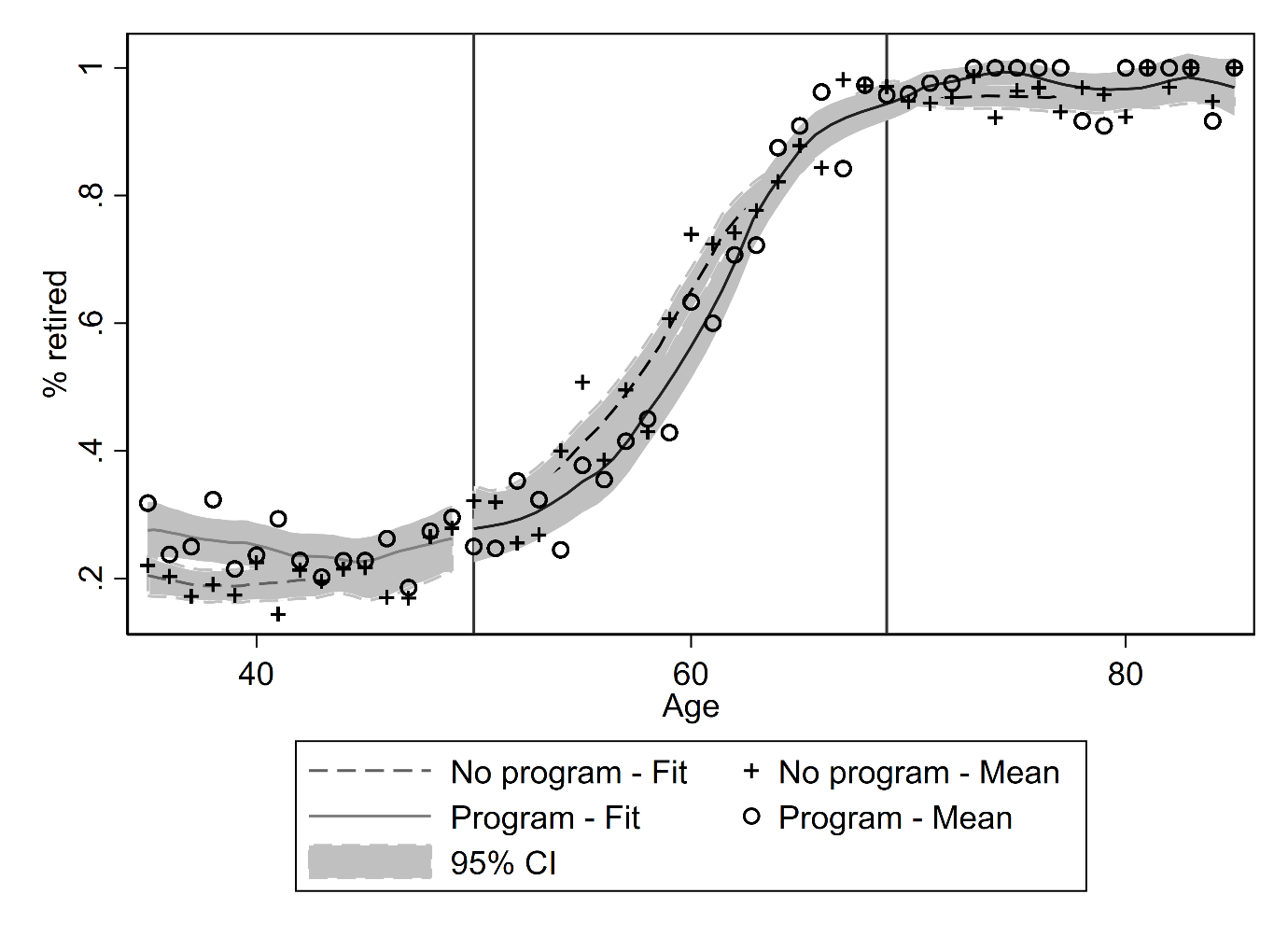
 Source: Eurobarometer, own calculations. The fitted lines show local mean fits of retirement against age. The vertical lines mark ages 50 and 69, the most common lower and upper limit for screening program eligibility in our sample. It should be noted that some countries offer screening from age 40 or 45 onwards, see Table A.1 in the online appendix.

## Figure C.3 Trends in mammography use by age


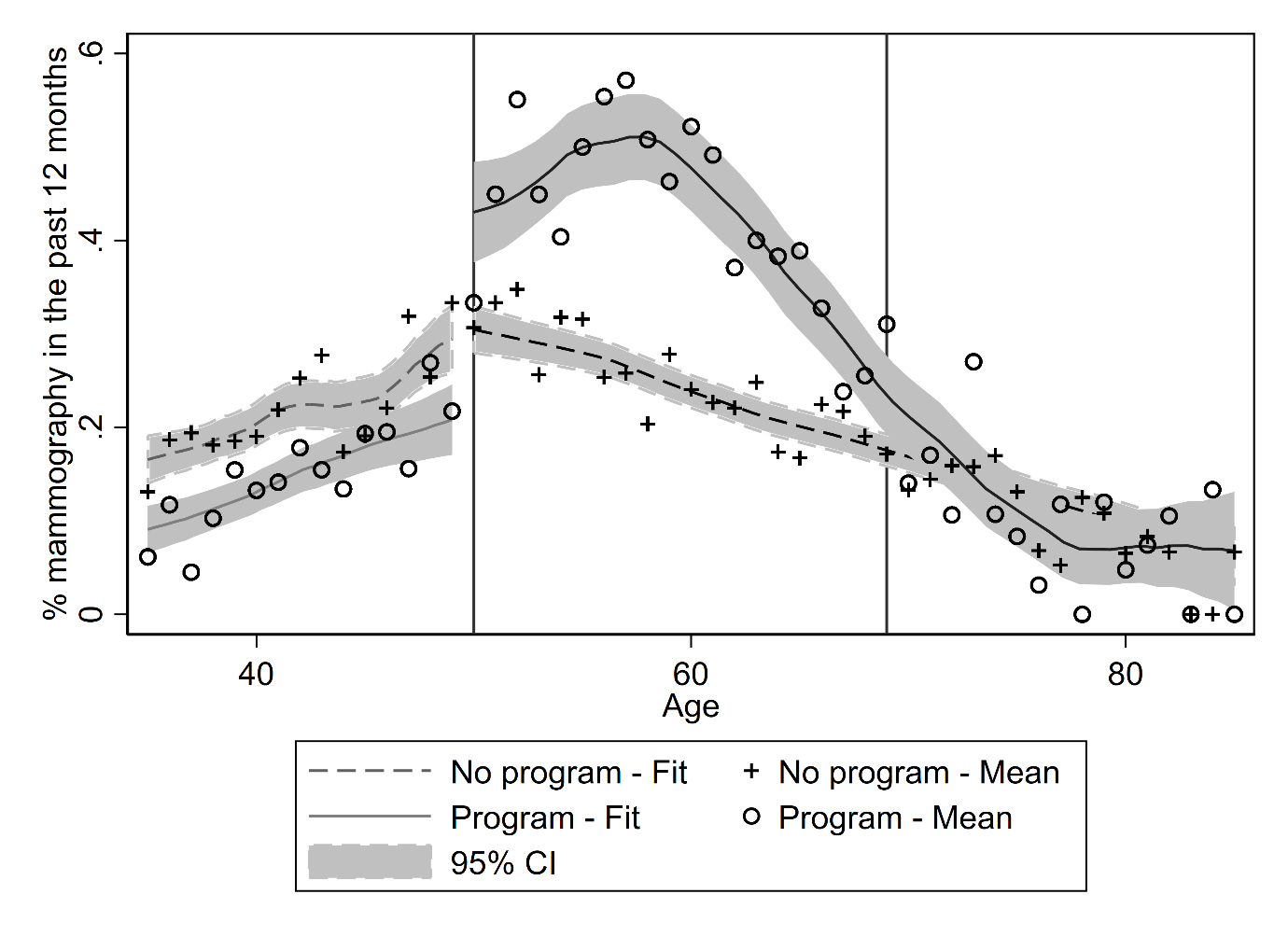


Source: Eurobarometer, own calculations. The fitted lines show local mean fits of mammography use in the past 12 months against age. The vertical lines mark ages 50 and 69, the most common lower and upper limit for screening program eligibility in our sample. It should be noted that some countries offer screening from age 40 or 45 onwards, see Table A.1 in the online appendix.

| Table C.1: Excluding countries from the treatment group | | | | | | | | | |
| --- | --- | --- | --- | --- | --- | --- | --- | --- | --- |
|  | **Excl. Finland** |  | **Excl. UK** |  | **Excl. Luxembourg** |  | **Excl. Netherlands** |  | **Excl. Sweden** |
| **Program** | 0.473*** |  | 0.495*** |  | 0.488*** |  | 0.489*** |  | 0.478*** |
|  | (0.076) |  | (0.077) |  | (0.074) |  | (0.076) |  | (0.063) |
| **Age range** | -0.026 |  | -0.038 |  | -0.008 |  | -0.024 |  | -0.019 |
|  | (0.043) |  | (0.044) |  | (0.043) |  | (0.043) |  | (0.043) |
| **Program x Age range** | 0.160*** |  | 0.131** |  | 0.135*** |  | 0.136** |  | 0.156*** |
|  | (0.052) |  | (0.053) |  | (0.049) |  | (0.053) |  | (0.049) |
|  |  |  |  |  |  |  |  |  |  |
| *N* | *9,925* |  | *9,759* |  | *10,205* |  | *9,964* |  | *9,958* |
| Source: Eurobarometer, own calculations. The estimates come from a linear model controlling for a quadratic age trend, education in four categories, country- and year-fixed effects. Standard errors clustered by country and age in parentheses. * p<0.1, ** p<0.05, *** p<0.01. | | | | | | | | | |

# Regional-level Analysis

We matched data on the implementation of regional programs with the NUTS-2 region indicators available in our data. Information on the existence of regional programs was derived from the following sources:

- Bastos et al. (2010)
- Willems and Bracke (2018)
- Goossens et al. (2014)
- Biesheuvel et al. (2011)
- Domingo et al. (2013)
- Ascunce et al. (2010)
- Chkotua and Peleteiro (2017)
- *Report of the Board of the National Cancer Screening Service January 2008-March 2010* (2010)
- *Primo Rapporto dell’Osservatorio Nazionale per la Prevenzione dei Tumori Femminili* (2002)
- Buchmueller and Goldzahl (2018)

We re-estimate our models using indicators for program existence and age range based on regional programs rather than country-wide programs. For countries that had already implemented a nationwide program and in which there was no variation in the age range across regions, these indicators were defined on the country-level. We also adjusted the model by including region-fixed effects rather than country-fixed effects, and by clustering standard errors by region and age (rather than country and age). We replicate our results shown in Table 2 (main model) and Table 4 (robustness checks) here. Replications of all other tables in the main text and the appendix with data on regional screening programs are available upon request.

We observe that the results are similar, but less precisely estimated. This might be due to the mismatch between program regions and NUTS-2 regions, e.g. in Italy and France, introducing measurement error into the estimation. It is also possible that nationwide programs use not only invitation letters but also other channels to provide information to the eligible population, e.g., mass media campaigns.

| **Table D.1: The effect of screening eligibility on health knowledge in regional programs** | | | | | | | | | | | | | | | | | |  |
| --- | --- | --- | --- | --- | --- | --- | --- | --- | --- | --- | --- | --- | --- | --- | --- | --- | --- | --- |
|  | **Main Specification** |  | **Additional covariates** |  | **Region-by-year FE** |  | **Cubic age trend** |  | **5-year age groups** |  | **No age controls** |  | **Without older control group** |  | **Alternative Scoring Method** |  | **Without women in the first interval** |  |
| **Program** | -0.135 |  | -0.051 |  | -0.757 |  | -0.142 |  | -0.138 |  | -0.126 |  | 0.036 |  | -0.087 |  | -0.062 |  |
|  | (0.365) |  | (0.359) |  | (0.720) |  | (0.365) |  | (0.364) |  | (0.377) |  | (0.397) |  | (0.561) |  | (0.387) |  |
| **Age range** | -0.041 |  | -0.041 |  | -0.03 |  | -0.035 |  | -0.017 |  | -0.012 |  | -0.024 |  | -0.074 |  | -0.067 |  |
|  | (0.046) |  | (0.046) |  | (0.045) |  | (0.047) |  | (0.062) |  | (0.037) |  | (0.053) |  | (0.058) |  | (0.049) |  |
| **Program x Age range** | 0.117** |  | 0.113** |  | 0.104** |  | 0.120** |  | 0.123** |  | 0.094* |  | 0.132*** |  | 0.135** |  | 0.143*** |  |
|  | (0.048) |  | (0.048) |  | (0.048) |  | (0.048) |  | (0.048) |  | (0.049) |  | (0.050) |  | (0.061) |  | (0.050) |  |
|  |  |  |  |  |  |  |  |  |  |  |  |  |  |  |  |  |  |  |
| *N* | *10,610* |  | *10603* |  | *10,610* |  | *10,610* |  | *10,610* |  | *10,610* |  | *9,006* |  | *10,610* |  | *10,023* |  |
| Source: Eurobarometer, own calculations. The estimates come from a linear model controlling for a quadratic age trend, education in four categories, region- and year-fixed effects. Standard errors clustered by region and age in parentheses. The additional covariates in column 2 are occupation (looking after home or family, student, unemployed, retired, farmer, professional, show owner/craftsmen, business proprietor, employed professional, general management, middle management, employed at a desk, employed travelling, employed service job, supervisor, skilled manual worker, unskilled manual worker), marital status (single, married, living as married, divorced, separated, widowed), household income in country-specific quartiles (incl. a category for missing values), whether childre under 15 are present in the household and whether other persons were present during the interview. Column 7 excludes women below the upper age limit for screening eligibility. In column 8, the health knowledge index is assigned 1 point for a "true" answer, 0 points for "don't know" and -1 point for "false" answers. * p<0.1, ** p<0.05, *** p<0.01. | | | | | | | | | | | | | | | | | |  |
|  |  |  |  |  |  |  |  |  |  |  |  |  |  |  |  |  |  |  |
|  |  |  |  |  |  |  |  |  |  |  |  |  |  |  |  |  |  |  |

# References for the Appendix

Althuis, M.D., Dozier, J.M., Anderson, W.F., Devesa, S.S., Brinton, L.A., 2005. Global trends in breast cancer incidence and mortality 1973–1997. International Journal of Epidemiology 34, 405–412. https://doi.org/10.1093/ije/dyh414

Ascunce, N., Salas, D., Zubizarreta, R., Almazán, R., Ibáñez, J., Ederra, M., representatives of the Network of Spanish Cancer Screening Programmes (Red de Programas Españoles de Cribado de Cáncer), 2010. Cancer screening in Spain. Annals of Oncology 21, iii43–iii51. https://doi.org/10.1093/annonc/mdq085

Bastos, J., Peleteiro, B., Gouveia, J., Coleman, M.P., Lunet, N., 2010. The state of the art of cancer control in 30 European countries in 2008. International Journal of Cancer 126, 2700–2715. https://doi.org/10.1002/ijc.24963

Biesheuvel, C., Weigel, S., Heindel, W., 2011. Mammography Screening: Evidence, History and Current Practice in Germany and Other European Countries. Breast Care (Basel) 6, 104–109. https://doi.org/10.1159/000327493

Bray, F., McCarron, P., Parkin, D.M., 2004. The changing global patterns of female breast cancer incidence and mortality. Breast Cancer Res 6, 229–239. https://doi.org/10.1186/bcr932

Buchmueller, T.C., Goldzahl, L., 2018. The effect of organized breast cancer screening on mammography use: Evidence from France. Health Economics 27, 1963–1980. https://doi.org/10.1002/hec.3813

Carrieri, V., Wuebker, A., 2016. Quasi-Experimental Evidence on the Effects of Health Information on Preventive Behaviour in Europe. Oxford Bulletin of Economics and Statistics 78, 765–791.

Chkotua, S., Peleteiro, B., 2017. Mammography Use in Portugal: National Health Survey 2014. Preventing Chronic Disease 14, 170054.

Domingo, L., Jacobsen, K.K., von Euler-Chelpin, M., Vejborg, I., Schwartz, W., Sala, M., Lynge, E., 2013. Seventeen-years overview of breast cancer inside and outside screening in Denmark. Acta Oncologica 52, 48–56. https://doi.org/10.3109/0284186X.2012.698750

Goossens, M., Van Hal, G., Van der Burg, M., Kellen, E., Van Herck, K., De Grève, J., Martens, P., Van Limbergen, E., 2014. Quantifying independent risk factors for failing to rescreen in a breast cancer screening program in Flanders, Belgium. Preventive Medicine 69, 280–286. https://doi.org/10.1016/j.ypmed.2014.10.019

Hicks, R., Tingley, D., 2011. Causal mediation analysis. Stata Journal 11, 605–619.

Imai, K., Keele, L., Tingley, D., 2010a. A general approach to causal mediation analysis. Psychological Methods 15, 309–334. https://doi.org/10.1037/a0020761

Imai, K., Keele, L., Yamamoto, T., 2010b. Identification, Inference and Sensitivity Analysis for Causal Mediation Effects. Statist. Sci. 25, 51–71. https://doi.org/10.1214/10-STS321

Imai, K., Tingley, D., Yamamoto, T., 2013. Experimental designs for identifying causal mechanisms. Journal of the Royal Statistical Society. Series A (Statistics in Society) 176, 5–32.

Jørgensen, K.J., Gøtzsche, P.C., 2006. Content of invitations for publicly funded screening mammography. BMJ 332, 538. https://doi.org/10.1136/bmj.332.7540.538

Primo Rapporto dell’Osservatorio Nazionale per la Prevenzione dei Tumori Femminili, 2002.

Report of the Board of the National Cancer Screening Service January 2008-March 2010, 2010.

Willems, B., Bracke, P., 2018. The impact of regional screening policies on the diffusion of cancer screening participation in Belgium: time trends in educational inequalities in Flanders and Wallonia. BMC Health Services Research 18, 943. https://doi.org/10.1186/s12913-018-3746-x
